# A novel functional interplay between Progesterone Receptor-B and PTEN, *via* AKT, modulates autophagy in breast cancer cells

**DOI:** 10.1111/jcmm.12363

**Published:** 2014-09-12

**Authors:** Francesca De Amicis, Carmela Guido, Marta Santoro, Marilena Lanzino, Salvatore Panza, Paola Avena, Maria Luisa Panno, Ida Perrotta, Saveria Aquila, Sebastiano Andò

**Affiliations:** a Centro Sanitario, University of CalabriaArcavacata di Rende (CS), Italy; b Department of Pharmacy, Health Science and Nutrition, University of CalabriaArcavacata di Rende (CS), Italy; c Post-graduate School in Clinical Pathology, University of CalabriaArcavacata di Rende (CS), Italy; d Di.B.E.S.T, University of CalabriaArcavacata di Rende (CS), Italy

**Keywords:** progesterone, PR-B, PTEN, AKT, breast cancer, autophagy, cell survival

## Abstract

The tumour suppressor activity of the phosphatase and tensin homologue on chromosome 10 (PTEN) is subject of intense investigative efforts, although limited information on its regulation in breast cancer is available. Herein, we report that, in breast cancer cells, progesterone (OHPg), through its cognate receptor PR-B, positively modulates PTEN expression by inducing its mRNA and protein levels, and increasing PTEN-promoter activity. The OHPg-dependent up-regulation of PTEN gene activity requires binding of the PR-B to an Sp1-rich region within the PTEN gene promoter. Indeed, ChIP and EMSA analyses showed that OHPg treatment induced the occupancy of PTEN promoter by PR and Sp1 together with transcriptional coactivators such as SRC1 and CBP. PR-B isoform knockdown abolished the complex formation indicating its specific involvement. The OHPg/PR-B dependent induction of PTEN causes the down-regulation of PI3K/AKT signal, switching on the autophagy process through an enhanced expression of UVRAG and leading to a reduced cell survival. Altogether these findings highlight a novel functional connection between OHPg/PR-B and tumour suppressor pathways in breast cancer.

## Introduction

Breast cancer accounts for over 20% of all cancer cases in women. Among breast cancer risk factors, overexposure to exogenous or endogenous estrogens has been widely assessed, while the role of progesterone (OHPg) is more controversial 1.

The effects of OHPg are mediated by the progesterone receptor (PR), two isoforms of which, PR-A and PR-B do exist and regulate different subsets of genes exhibiting distinct roles 2–4. *In vivo* the two PR isoforms are usually co-expressed at similar levels in normal cells but their ratio varies dramatically in different tissues, physiological states and disease sites 5,6. Specifically the ratio between PR-A and PR-B is increased in breast tumours from patients with poor prognosis, with a predominance of PR-A and loss of PR-B 7.

Recently it has been reported that, in rat brain, progesterone regulates the expression of Phosphatase and Tensin homolog deleted on chromosome TEN (PTEN) gene 8. PTEN acts as a tumour suppressor by dephosphorylating the plasma membrane lipid second messenger phosphoinositide-3,4,5-trisphosphate (PIP3) antagonizing signal transduction downstream of phosphatidylinositol-3 kinase (PI3K) and suppressing cell growth and survival 9. PTEN germline mutations have been associated with increased risk of breast cancer and reduced or absent PTEN protein expression has been recognized in up to 50% of breast tumours 9.

PTEN has also been shown to control autophagy in mammalian cells through its lipid phosphatase activity which antagonizes the inhibitory effect of the PI3K/AKT pathway on the autophagic sequestration that involves type III PI3-kinase 10. However, limited information on the regulation of PTEN expression in breast cancer is available and the possible functional link between PR and PTEN in the breast has not been evaluated, yet.

In the present study, we provide evidence that PTEN might be at least one of the potential effector through which PR-B exerts its protective effects in breast cancer cells. We demonstrated that OHPg/PR-B up-regulates PTEN expression which in turn, through the inhibition of PI3K/AKT pathway, allows an enhanced expression of UVRAG, leading to a reduced cell survival because of autophagy.

## Materials and methods

### Reagents

17-Hydroxyprogesterone (OHPg), aprotinin, leupeptin, phenylmethylsulfonyl fluoride (PMFS), sodium orthovanadate, NaCl, MgCl_2_, EGTA, glycerol, Triton X-100, charcoal-treated foetal calf serum (FCS), HEPES, insulin-like growth factor1 (IGF1) Mithramycin A and 3-Methyladenine (3-MA) were from Sigma-Aldrich (Milan, Italy). Antibodies against human PTEN*,* PR, β-actin, pAKT1/2/3, AKT1/2/3, ultraviolet irradiation resistance associated tumour suppressor gene (UVRAG), and Protein A/G PLUS-Agarose were from Santa Cruz Biotechnology (Santa Cruz, CA, USA). Antibodies to CBP-related protein p300 (CBP), steroid receptor coactivator 1 (SRC1), mTOR, PI3 Kinase Class III and Beclin-1 (Beclin1) were from Cell Signaling (Beverly, MA, USA).

### Plasmids

The firefly luciferase reporter plasmid containing the full-length of the PTEN promoter region [pGL3-2768 (-2927/-160)] and the different deletion constructs [pGL3-612 (-1389/-778), pGL3-341 (-1118/-778), pGL3-139 (-916/-778)] gifts from Prof. Xi-Liang Zha (Shanghai Medical College, Fudan University, Shanghai) 11. The full-length PR-B consisting of the full-length PR-B cDNA fused with the SV40 early promoter and expressed in the pSG5 vector gift from Dr. D. Picard (University of Geneva, Switzerland); the full-length PR-A provided by Prof. Paul Kastener (Laboratary of Moleculare Genetic, CNRS, Strasbourg, France) 12. The expression plasmids of Akt kinase (myristoylated AKT) encoding constitutive active forms were provided from Drs. P. Tsichlis and T. Chan (Kimmel Cancer Center-Philadelphia). The Renilla luciferase expression vector pRL-TK (Promega, Milan, Italy) was used as a transfection standard.

### Cell culture

Human breast cancer MCF-7 cells, T47D human breast cancer cells and human uterine cervix adenocarcinoma (HeLa) cells were obtained from the American Type Culture Collection (ATCC, Manassas, VA (USA)). MCF-7 and HeLa cells were maintained in DMEM/F-12 medium containing 5% FCS, 1% L-glutamine, 1% Eagle’s nonessential amino acids and 1 mg/ml penicillin/streptomycin in a 5% CO_2_ humidified atmosphere. T47D cells were routinely maintained in RPMI 1640 supplemented with 5% FCS, 1 μg/ml insulin (Sigma-Aldrich), 1 mg/ml penicillin/streptomycin (Sigma-Aldrich). Cells were cultured in phenol red-free medium, 0.5% bovine serum albumin and 2 mM L-glutamine (serum-free medium), for 48 hrs before each experiment. Hormone stimulation was performed in medium containing 1% charcoal-treated FCS to reduce the endogenous steroid concentration 13.

### Reverse transcription and real-time PCR

Cells were treated as indicated and processed as previously described 14. The primers all from Invitrogen were: (PTEN forward) 5′-CCACCACAGCTAGAACTTATC-3′; (PTEN reverse) 5′-ATCTGCACGCTCTATACTGC-3′.

### Western blotting and immunoprecipitation

MCF-7 cells were grown in 10 cm dishes to 70–80% confluence, exposed to treatments for 24 hrs and lysed. Total or nuclear extracts were prepared and subjected to SDS-PAGE as previously described 15.

For immunoprecipitation 16, 500 μg of protein lysates were incubated overnight with the specific antibody and 500 μl of HNTG buffer [50 mmol/l HEPES (pH 7.4), 50 mmol/l NaCl, 0.1% Triton X-100, 10% glycerol, 1 mmol/l PMFS, 10 Ag/ml leupeptin, 10 Ag/ml aprotinin]. Immunocomplexes were recovered by incubation with protein A/G-agarose. The beads containing bound proteins were washed by centrifugation in immunoprecipitation buffer then denatured by boiling in Laemmli sample buffer and analysed by Western blot. The images were acquired by using an Epson Perfection scanner (Epson, Tokyo, Japan) using Photoshop software (Adobe, San José (CA) USA). The optical densities of the spots were analysed by using ImageJ software (NIH; http://rsb.info.nih.gov/IJ).

### Transfections and luciferase assays

Transfections were done as described 17 using Fugene 6 reagent (Roche Diagnostics, Milan, Italy). Luciferase activity was measured with the Dual Luciferase kit (Promega).

### Lipid-mediated transfection of siRNA duplexes

Custom-synthesized siRNA (Invitrogen, Paisley, UK), annealed duplexes (25-bp double-stranded RNA [dsRNA]) were used for effective depletion of PTEN and PR-B. A non-specific siRNA (Invitrogen) was used for non-sequence-specific effects. Cells were transfected using Lipofectamine 2000 reagent (Invitrogen) as previously reported 18 and then treated as indicated.

### Chromatin immunoprecipitation (ChIP) assays

Cells were treated as indicated before harvesting for the assay performed as described 19. PTEN promoter primers, corresponding to the region between −1118 bp to −916 bp, used for PCR: forward 5′-ATGCTCAGTAGAGCCTGCGGCTT-3′ and reverse 5′-AAGACCGAGGGGAGGCGGGAA-3′ were from Invitrogen.

### Electrophoretic mobility shift assay (EMSA)

Electrophoretic mobility shift assay was carried out as previously described 20. Cells were treated as indicated before harvesting for the assay. The DNA sequence used as probe or as cold competitor was 5′-CTCCTACCGCCCCCTGCCCTGCCCT-3′ from Invitrogen.

### Cell survival analysis

MCF-7 cell survival analysis was performed as previously described 16 using trypan blue exclusion assay (0.2% trypan blue). Cells were seeded in 12-well plates in regular growth medium, processed as requested and then incubated with the indicated treatments; after 48 hrs cells were trypsinized and counted in a hemocytometer under phase contrast microscope. The results were expressed as percentage of the controls, determined by standardizing untreated cells to 100%.

### Soft agar growth assay

Anchorage-independent growth assays were conducted as previously described 21. Data represent the mean of three independent experiments carried out in triplicate.

### Acidic vesicular organelle staining

The formation of acidic compartments was quantified by flow cytometric analysis of acridine orange stained cells. The intensity of the red fluorescence is proportional to the amount of acidity.

Following treatment, cells were stained with 1 mg/ml acridine orange for 15 min. at 37°C. Cells were then trypsinized and collected in phenol red-free medium. Green (510–530 nm) and red (>650 nm) fluorescence emission from cells was measured with a Fluorescence Activated Cell Sorter (FACS) from Becton Dickinson, Franklin Lakes, NJ, USA.

### Transmission electron microscopy

Transmission electron microscopy was conducted as previously described 22. Briefly, cells treated as indicated were fixed in 3% glutaraldehyde (Sigma-Aldrich) solution in 0.1 M phosphate buffer (pH 7.4) for 2 hrs. Then the samples were post-fixed in osmium tetroxide (3%), dehydrated in graded acetone and embedded in Araldite (Sigma-Aldrich). Ultrathin sections were collected on copper grids and contrasted using both lead citrate and uranyl acetate. The grids were examined in a ‘Zeiss EM 10’ electron microscope. TEM images were also used to count and calculate the number of autophagic vacuoles per cross-sectioned cell by three independent investigators. Per field, 50 cells were counted and ten different fields were analysed per sample 23.

### Statistical analysis

The statistical analysis of experimental data was performed with a Student’s *t*-test (comparison of two groups) or one-way anova (comparison of more than two groups) were appropriate and results were presented as mean ± SD. A value of *P* ≤ 0.05 was considered to be significant.

## Results

### OHPg through the PR-B isoform induces PTEN expression in breast cancer cells

**Figure 1:**
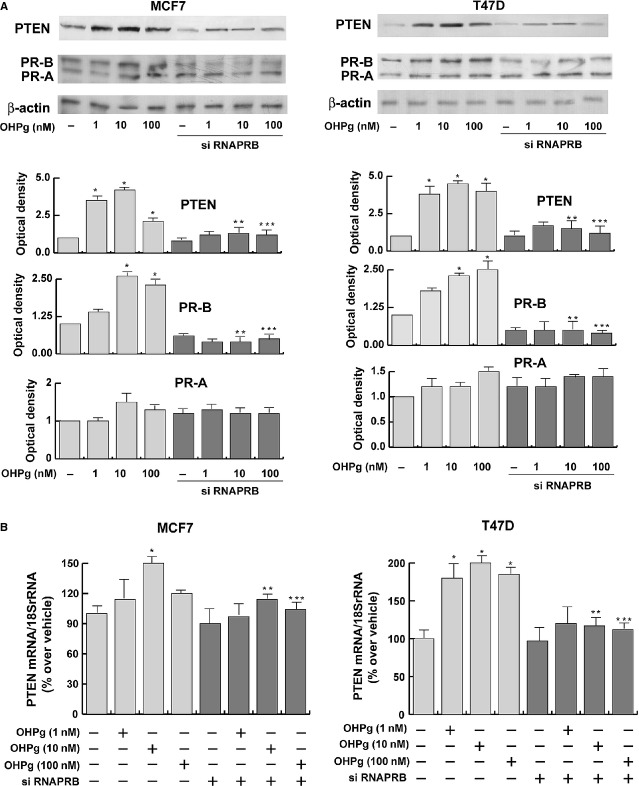
OHPg through PR-B up-regulates PTEN protein and mRNA levels in breast cancer cells. (**A**) *Upper panel*, Western blot analysis of PTEN and PR expression in MCF-7 and T47D breast cancer cells transfected with non-specific or targeted against PR-B siRNA and treated with increasing concentration of OHPg, as indicated. β-actin was used as loading control. Autoradiographs show the results of one representative experiment. Columns, are mean of three independent experiments in which band intensities were evaluated in terms of optical density arbitrary units and expressed as fold over vehicle, which was assumed to be 1; bars, SD;*P < 0.05 *versus* vehicle treated cells. **P < 0.05 *versus* 10 nM OHPg. ***P < 0.05 *versus* 100 nM OHPg. (**B**) Real-time PCR assay of *PTEN* mRNA expression. MCF-7 and T47D cells were transfected with non-specific- or targeted against PR-B siRNA and treated for 24 hrs with vehicle (−) or OHPg, as indicated. 18S mRNA was determined as control. Columns are the mean of three independent experiments each in triplicate; bars, SD; *P < 0.05 *versus* vehicle treated cells; **P < 0.05 *versus* 10 nM OHPg; ***P < 0.05 *versus* 100 nM OHPg.

First, we evaluated the ability of OHPg to modulate *PTEN* expression in MCF-7 and T47-D breast cancer cell lines, which express wild-type PTEN 24. After 24 hrs of treatment, OHPg at all tested concentrations, induced a significant increase in PTEN expression in terms of both protein and mRNA levels, as assessed by Western blotting (Fig. 1A) and Real-time PCR (Fig. 1B). To investigate which one of the two PR isoforms mediates the OHPg up-regulatory action on PTEN expression, we performed PR-B siRNA knockdown experiments in MCF-7 and T47D cells. Addition of a PR-B-targeting siRNA resulted in reduction in PR-B protein levels (Fig. 1A) and clearly abrogated the OHPg-dependent up-regulation of PTEN protein and mRNA levels, indicating the involvement of PR-B in the modulation of PTEN expression.

### OHPg up-regulates PTEN gene promoter activity

**Figure 2:**
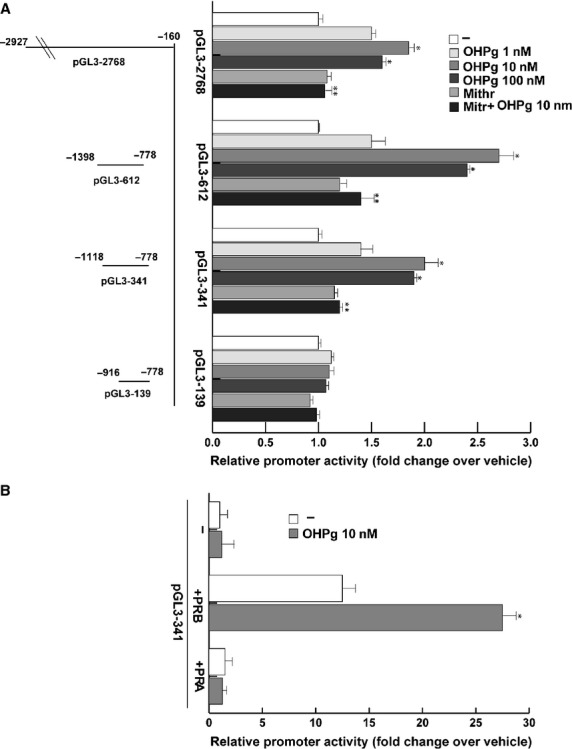
OHPg transactivates PTEN promoter gene in MCF-7 cells. (**A**) *left panel*. Schematic representation of deletion fragments of the PTEN gene promoter. *Right panel*. Constructs depicted were transiently transfected in MCF-7 cells, treated with vehicle (−) or OHPg and/or Mithr, as indicated. (**B**) HeLa cells were cotransfected with an empty vector or with the PTEN promoter construct pGL3-341 and PR-B or PR-A expression vector, and then treated for 24 hrs with vehicle (−) or 10 nM OHPg. After 24 hrs, cells were harvested and luciferase activities were determined. Columns are mean of three independent experiments and expressed as fold change over vehicle, which was assumed to be 1; bars, SD; *P < 0.05 *versus* vehicle. **P < 0.05 *versus* 10 nM OHPg.

To evaluate whether OHPg/PR-B could modulate PTEN promoter gene transcriptional activity, we performed transient transfection assays in MCF-7 cells, by using a full-length PTEN gene promoter expression vector and a series of PTEN promoter deleted constructs (Fig. 2A, *left panel*) previously described as an useful tool to study the regulation of PTEN expression 11,25. As shown in Figure 2A, *right panel*, OHPg significantly increased the activity of constructs pGL3-2768, pGL3-612 and pGL3-341. On the contrary, OHPg failed to enhance the transcriptional activity of the PTEN promoter when cells were transfected with pGL3-139 construct, indicating that the region between −1118 bp and −916 bp was necessary for the up-regulation of PTEN promoter activity by OHPg. Similar results were obtained in T47D cells (data not shown).

Sequence analysis, showed that this DNA region does not contain any canonical progesterone responsive element (PRE) or AP-1 binding sites. On the other hand, this region contains several DNA motifs binding Sp1 26, a transcription factor which can interact with PRs to modulate transcription of specific genes 27,28. To assess Sp1 involvement in the OHPg/PR-dependent induction of PTEN gene activity, MCF-7 cells were treated with Mithramycin (Mithr) a drug able to inhibit Sp1-DNA binding 29. As shown in Figure 2A, *right panel*, 100 nM Mithr administration prevented the transactivation of PTEN promoter by OHPg.

Progesterone receptor-B specific involvement in the modulation of PTEN promoter gene activity was proved in PR-negative HeLa cells cotransfected with expression plasmids encoding either PR-B or PR-A (Fig. 2B). PR-B isoform expression itself was able to markedly increase the activity of PTEN promoter which was further enhanced by OHPg treatment. Instead, ectopic expression of PR-A isoform did not exert any significant effect. Altogether, these data strongly suggest that PR-B/Sp1complex formation is fundamental for transcriptional regulation of PTEN by OHPg.

### PR-B associates with Sp1 on PTEN promoter after OHPg treatment in MCF-7 cells

**Figure 3:**
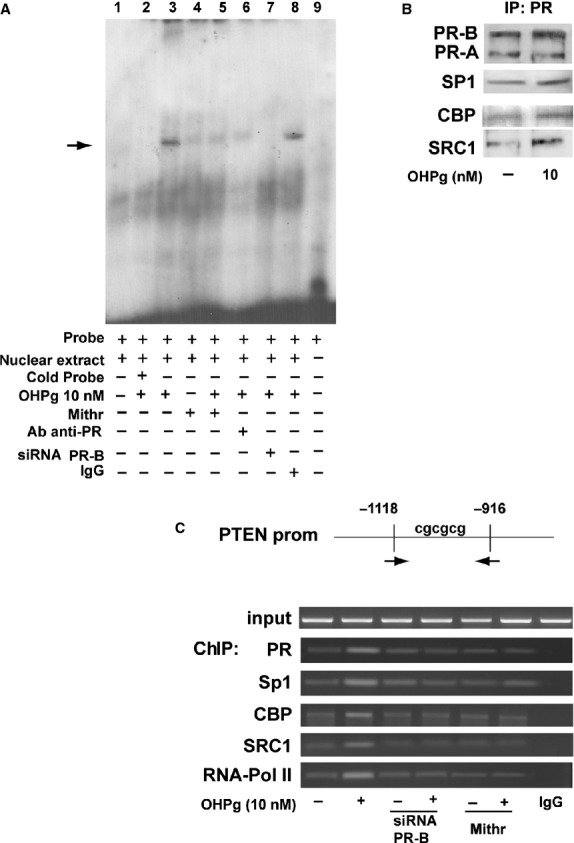
Ligand-activated PR-B, through SP1, binds the PTEN gene promoter. (**A**) Electrophoretic mobility shift assay on nuclear extracts from MCF-7 cells untreated (*lane 1*) or treated with 10 nM OHPg for 2 hrs (*lane 2,3,5-8*). 100-fold molar excess of unlabelled probe (*lane 2*); addition of 100 nM Mithr (*lane 4,5*); pre-incubation with anti-PR antibody (*lane 6*); PR-B silencing by specific siRNA (*lane 7*); pre-incubation with anti-IgG antibody (*lane 8*); probe alone (*lane 9*). (**B**) Co-immunoprecipitation analysis of PR-associated proteins. Nuclear extracts from MCF-7 cells vehicle treated (−) or 10 nM OHPg for 24 hrs, were immunoprecipitated by using specific anti-PR antibody and filter was blotted with anti-PR, or -SP1 or-CBP or -SRC1 antibodies. (**C**) Sheared chromatin from MCF-7 cells transfected with non-specific- or targeted against PR-B siRNA and treated for 2 hrs with vehicle (−) or OHPg or Mithr as indicated, was precipitated using anti-PR or -SP1, or -CBP, or -SRC1, or -RNA Pol II antibodies. IgG, control samples. DNA Input, loading control. Results are representative of three independent experiments.

To better investigate the PR interaction within PTEN gene promoter, MCF-7 cells were treated or not with 10 nM OHPg. Cell nuclear extracts were prepared and used in EMSA assay using a synthetic oligodeoxyribonucleotide containing the identified OHPg-responsive sequence of the PTEN promoter bearing the Sp1 binding motifs (Fig. 3A). We observed the formation of a specific complex (*lane 1*) which was increased upon 10 nM OHPg treatment (*lane 3*) and abrogated by a 100-fold molar excess of unlabelled probe (*lane 2*), demonstrating the specificity of the DNA-binding complex. To confirm the Sp1 involvement in the formation of the detected protein-DNA complexes, nuclear extracts from cells co-treated with 100 nM Mithr were used, evidencing a strong reduction in the complex formation (*lane 5*). The involvement of PR in the DNA-binding complexes was confirmed by pre-incubation with an anti-PR antibody (*lane 6*) which caused immune-depletion of the protein-DNA complex. Specific PR-B siRNA confirmed the presence of this isoform in the complex (*lane 7*). Finally, normal rabbit IgG addition did not affect protein–DNA complex formation (*lane 8*).

To gain further insight into the mechanism involved in the PTEN transactivation induced by OHPg, we performed immunoprecipitation experiments. As shown in Figure 3B, OHPg administration enhanced interaction of PR with Sp1 as well as with the transcriptional coactivators CBP and SRC1. These results support the hypothesis that a complex among PR, Sp1, CBP and SRC1 does exist. To assess the contribution of this protein complex to the induction of PTEN gene expression *in vivo*, we performed a ChIP assay (Fig. 3C). MCF-7 cells were treated with 10 nM OHPg and protein-DNA complexes were immunoprecipitated with antibodies directed against PR, Sp1, CBP, SRC1 and RNA polymerase II. Obtained results demonstrated that OHPg treatment caused an enhanced recruitment of PR to the PTEN promoter together with an increased recruitment of Sp1, CBP and SRC1.

The concomitant increase in RNA-Pol II recruitment confirmed the positive regulation of PTEN transcriptional activity by OHPg. The role of the specific PR-B/Sp1 interaction within the PTEN promoter sequence in mediating its transcriptional activation was further strengthened by specific silencing of PR-B isoform or addition of 100 nM Mithr, since they both completely abrogated the increased recruitment of CBP and SRC1 as well as RNA-Pol II. PR recruitment did not occur on an unrelated PTEN promoter region located upstream of the −1118 bp to −916 bp region and not containing GC rich boxes (data not shown).

### OHPg/PR-B reduces cell survival by targeting PTEN in breast cancer cells

**Figure 4:**
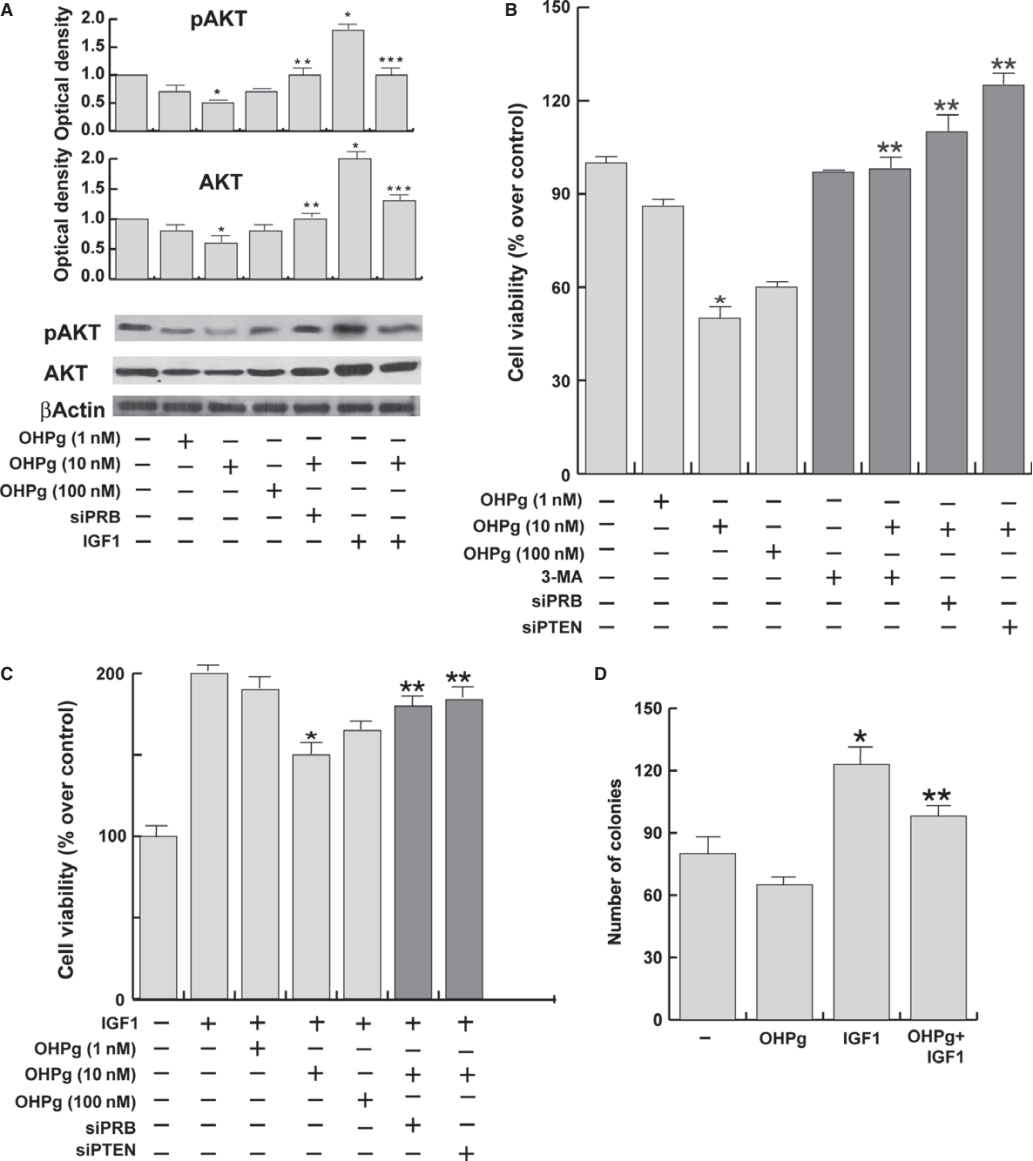
OHPg treatment reduces cell viability in MCF7 cells. (**A**) Western blot analysis of pAKT and total AKT expression. MCF-7 cells transfected with non-specific- or targeted against PR-B siRNA were treated for 24 hrs with vehicle (−) or OHPg and/or IGF1, as indicated. β-actin was used as loading control. Columns, are mean of three independent experiments in which band intensities were evaluated in terms of optical density arbitrary units and expressed as fold over vehicle, which was assumed to be 1; bars, SD;*P < 0.05 *versus* vehicle. **P < 0.05 *versus* 10 nM OHPg. ***P < 0.05 *versus* 50 nM IGF-1. (**B**) MCF-7 cells transfected with non-specific- or targeted against PR-B or PTEN siRNA were treated for 24 hrs with vehicle (−), OHPg and/or 5 mM 3-MA as indicated. Columns are mean of three independent experiments and expressed as % over vehicle, which was assumed to be 100%; bars, SD; *P < 0.05 *versus* vehicle. **P < 0.05 *versus* 10 nM OHPg. (**C**) MCF-7 cells transfected with non-specific- or targeted against PR-B or PTEN siRNA were treated for 24 hrs with vehicle (−) or IGF1 and/or OHPg, as indicated. Columns are mean of three independent experiments and expressed as % over vehicle, which was assumed to be 100%; bars, SD; *P < 0.05 *versus* vehicle. **P < 0.05 *versus* 10 nM OHPg. (**D**) Anchorage-independent cell growth assay in MCF-7 cells treated with vehicle (−) or with 10 nM OHPg and/or 50 nM IGF1. Columns are mean of three independent experiments; bars, SD; *P < 0.05 *versus* vehicle. **P < 0.05 *versus* 10 nM OHPg.

PTEN is known to negatively regulate the PI3K/AKT pathway thus affecting cell growth and survival in different cell types 9. Consistently, in our experimental conditions addition of 10 nM OHPg reduced the cellular content of pAKT and total AKT (Fig. 4A) and its downstream target mTOR (Fig. S1) either in basal conditions or following treatment with IGF-1, a relevant factor in breast cancer growth and progression 30. Therefore to better understand the biological significance of the OHPg/PR-B-dependent modulation of PTEN, we analysed breast cancer cell proliferation by using Trypan blue exclusion assay. Either 10 or 100 nM OHPg produced a significant reduction in MCF-7 basal (Fig. 4B) or IGF-1-stimulated cell proliferation (Fig. 4C). Specific silencing of PR-B or PTEN by siRNAs abrogated the inhibitory effect produced by OHPg on cell survival, suggesting that a molecular connection between OHPg/PR-B signalling and PTEN does exist and is fundamental in mediating OHPg-dependent effects.

In addition, 10 nM OHPg treatment also antagonized the IGF1-dependent MCF-7 colony formation in soft agar (Fig. 4D).

### PR-B and PTEN interplay mediates autophagy in breast cancer cells

In the last few years, an intriguing correlation between cell survival and autophagy process has been realized. Therefore, to determine whether autophagy occurrence might be related to the OHPg-dependent inhibition of cell survival, MCF-7 cells were co-treated with the autophagy inhibitor 3-Methyladenine (3-MA) which effectively blocked the OHPg effects (Fig. 4B).

Since recently an inverse relationship between AKT and UVRAG, a key factor in autophagosome formation has been recently demonstrated 31, we tested whether AKT could represent a molecular link between OHPg/PR-B/PTEN and autophagy in MCF-7 cells.

**Figure 5:**
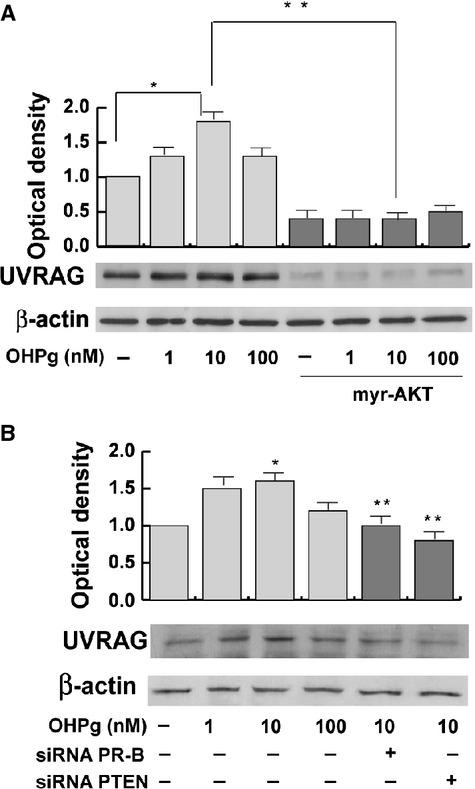
OHPg modulates the expression of proteins involved in autophagy process in MCF-7 cells. (**A**) MCF-7 cells transfected with an empty vector or with a constitutive active myristilated-AKT (myr-AKT) were treated for 24 hrs with vehicle (−) or increasing OHPg concentrations, as indicated. β-actin was used as loading control. Columns, are mean of three independent experiments in which band intensities were evaluated in terms of optical density arbitrary units and expressed as fold over vehicle, which was assumed to be 1; bars, SD; *P* < 0.05. (**B**) Western blot analysis of UVRAG expression. MCF-7 cells transfected with non-specific- or targeted against PR-B or PTEN siRNA were treated for 24 hrs with vehicle (−) or OHPg as indicated. β-actin was used as loading control. Columns, are mean of three independent experiments in which band intensities were evaluated in terms of optical density arbitrary units and expressed as fold over vehicle, which was assumed to be 1; bars, SD; *P < 0.05 *versus* vehicle treated cells. **P < 0.05 *versus* 10 nM OHPg.

To this aim MCF-7 cells were transfected with an empty vector or a constitutively active myristilated AKT and assessed for the capacity of OHPg to modulate UVRAG expression. As shown in Figure 5A and B OHPg treatment up-regulated UVRAG protein levels. Ectopic expression of the constitutively active AKT inhibited endogenous basal UVRAG expression and was able to completely abolish the OHPg effect on UVRAG cell content (Fig. 5A). Specific silencing of PR-B or PTEN by siRNAs abrogated the effect produced by OHPg (Fig. 5B).

**Figure 6:**
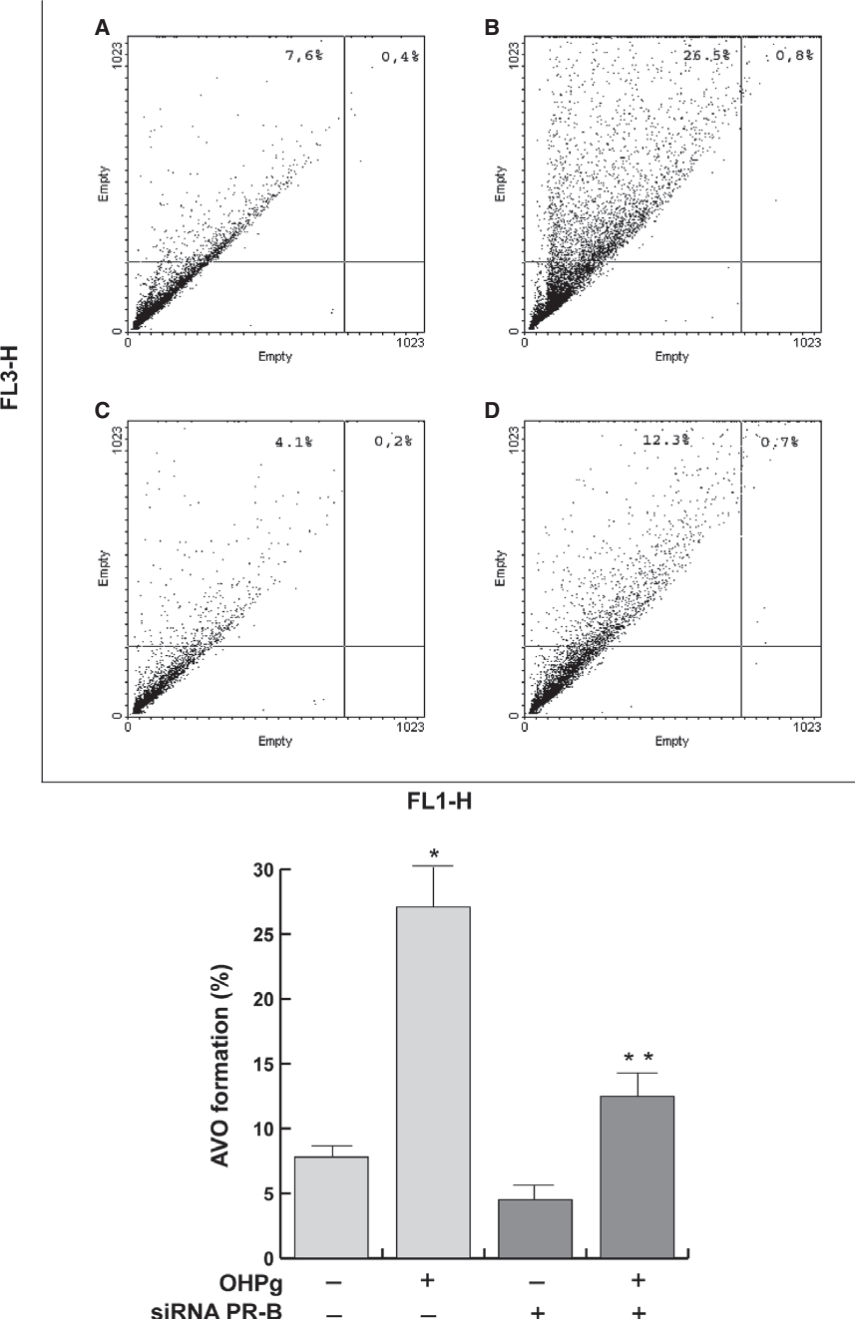
OHPg treatement increases acidic vesicular organelle (AVO) formation in MCF-7 cells. MCF-7 cells transfected with non-specific- (**A** and **B**) or targeted against PR-B siRNA (**C** and **D**) and treated for 24 hrs with vehicle (**A** and **C**) or OHPg (**B** and **D**), were incubated with acridine orange and fluorescence analysed by flow cytometry. Representative flow cytometry dot plots are shown. Columns are mean of three independent experiments; bars, SD; *P < 0.05 *versus* vehicle.

To screen for increased autophagic activity following OHPg treatment, we determined acidic vesicular organelle (AVO) formation by flow cytometry analysis of acridine orange stained MCF-7 cells, using the FL3 mode to measure the bright red fluorescence/AVO formation and the FL1 mode to measure the green fluorescence/uncharged acridine orange. As shown in Figure 6, after 24 hrs, OHPg increased AVO formation (from 8% to 27%) and PR-B siRNA transfection counteracted this effect.

**Figure 7:**
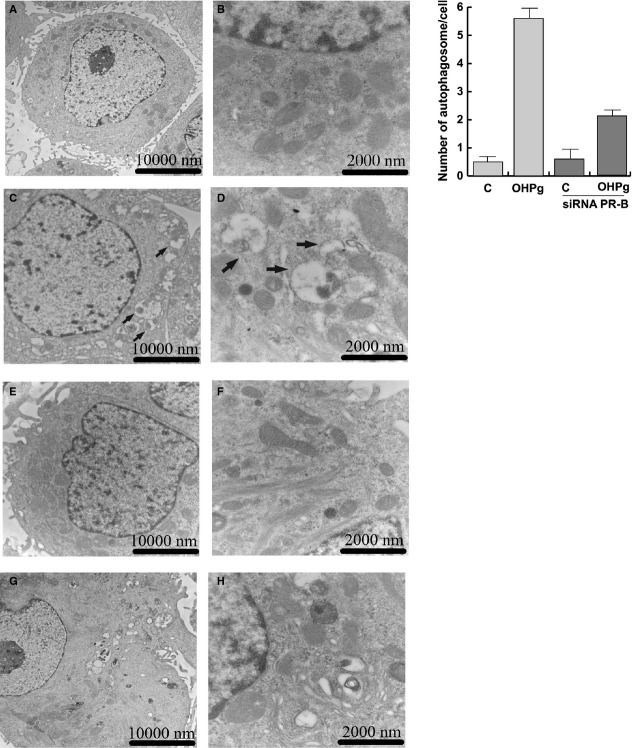
Autophagy was induced in MCF-7 breast cancer cells by OHPg treatement. TEM analysis (left panel) was conducted in MCF-7 cells (**A**–**D**) and in MCF-7 cells transfected with PR-B siRNA (**E**–**H**) treated with vehicle (−) (**A**, **B**, **E**, **F**) or 10 nM OHPg (**C**, **D**, **G**, **H**) for 24 hrs. Arrows indicate the autophagic bodies. Figure shows the results of one representative out of three independent experiments. Images magnification: **A** 6300X, **B** 31,500X, **C** 10,000X, **D** 31,500X, **E** 12,500X, **F** 25,000X, **G** 6300X, **H** 31,500X. Quantification of the number of autophagic bodies (right panel) per cross-sectioned cell. Per field, 50 cells were counted and ten different fields were analysed per sample.

Finally, the occurrence of an OHPg-dependent autophagic phenotype was monitored by transmission electron microscopy (TEM) 32. Ultrastructural analysis revealed that all the organelles, plasma membrane and nucleus of MCF-7 cells were well preserved in all the experimental condition tested (Fig. 7). OHPg treatment, induced the accumulation of autophagic vacuoles (Fig. 7C and D) and specific silencing of PR-B attenuated the effect produced by OHPg (Fig. 7G and H). The quantitative analysis of the number of autophagosome also showed a marked increase after OHPg administration. Consistently, OHPg through PR-B, significantly induced the expression of other fundamental autophagies, such as PI3KIII and Beclin 1 (Fig. S2).

## Discussion

Decreased PR-B expression has been related to breast tumour progression and treatment failure 3,7. Specifically, experimental models show that ER+/PR+ breast cancers are well differentiated, while ER+/PR− metastatic tumours display a much more aggressive course compared with tumours retaining PR 33. In the present article, we investigated whether ligand-activated PR-B could cooperate with PTEN by inducing autophagy in breast cancer cells.

PTEN takes part in controlling cellular processes including cell growth, spreading and migration 34. Despite extensive characterization of PTEN mutations in human cancers and relatively good understanding of the molecular role of PTEN in the control of cellular processes, little is known about the mechanisms of PTEN regulation.

Herein, we demonstrated that, in MCF-7 and T47D breast cancer cells, PR specific ligand OHPg determines an effective increase in both PTEN mRNA and protein levels. This effect is consistent with a previous study showing the ability of progesterone to increase PTEN expression in human endometrium 35.

OHPg modulation of PTEN expression occurs at transcriptional level as demonstrated by functional studies that also point out the fundamental role of activated PR-B in the up-regulation of PTEN cellular levels, since in PRs-negative HeLa cells induction of PTEN promoter activity can be demonstrated only in the presence of exogenous PR-B expression.

Interestingly, using deleted constructs of the PTEN gene promoter we identified a region spanning from −1118 bp to −916 bp which is responsive to OHPg. Analysis of this DNA sequence indicates the absence of any canonical PREs or AP-1 binding sites. Instead, several DNA motifs binding the Sp1 transcription factor are present. Indeed, inhibition of the Sp1-DNA binding, by co-treatment with Mithr, prevents the OHPg induced transactivation of PTEN gene promoter underlining the importance of Sp1 in the modulation of PTEN gene expression by OHPg. Our results are in agreement with previous studies reporting that Sp1 is a regulator of PTEN expression and that it can mediate PR-dependent gene transcription 28,36.

From our study, it emerges that modulation of the activity of the human PTEN promoter is consequent to the contemporary binding of PR-B and Sp1 to the identified OHPg-responsive sequence, as indicated by EMSA and ChIP assays. The stimulatory role of OHPg/PR-B/Sp1 on PTEN gene promoter, is demonstrated by the dynamic of RNA Pol II recruitment, that appears to be enhanced upon OHPg treatment. Different studies demonstrated that PR preferentially recruits the transcriptional coactivator SRC1 that was observed to act synergistically with CBP to enhance PR transactivation on a chromatin template *in vitro* 37. Moreover, SRC1 and CBP are known to associate both *in vitro* and *in vivo* and further analysis of their synergism revealed an obligatory ‘sequential’ recruitment to liganded PR 37,38. Consistent with these data, our ChIP experiments show that among the potential coactivators able to interact with PR, both SRC-1 and CBP are present with PR-B within the investigated PTEN gene promoter sequence. PR-B/SRC-1/CBP association increases upon OHPg treatment.

Overall, our data indicate for the first time that PR-B through Sp1 interacts with PTEN gene promoter and modulates its transcriptional activity, thus disclosing OHPg/PR-B as a new PTEN modulator.

The biological outcome of the functional interplay between PR-B and PTEN is represented by a the appearance of an autophagic phenotype which is crucial in the inhibition of breast cancer cell survival as demonstrated by results obtained using the autophagy inhibitor 3-MA. The decline in MCF-7 cell survival upon increasing OHPg concentrations is abrogated by PR-B as well as PTEN-knockdown, indicating that a co-operation between these two factors is needed to OHPg for reducing breast cancer cell viability.

One of the mechanisms through which PTEN exerts its role in tumour suppression is the interfering with the IGFs system 39. Notably, in our study, OHPg/PR-B/PTEN axis negatively influences the IGF-1 stimulatory effect on cell number and anchorage-independent cell growth.

Consistent with such a scenario, it was demonstrated that the synergistic effect of conditional PTEN loss and oncogenic K-ras mutation on endometrial cancer development occurs *via* decreased PR action 40. Therefore, we propose that efficient PR-B-mediated effects require PTEN action and that the PR-B/PTEN functional interplay has the ability to modulate cell survival signalling, extending the beneficial role of this nuclear receptor on breast cancer cells. Specifically our data support an inhibitory function of OHPg/PR-B/PTEN axis on PI3K/AKT pathway that has been recently demonstrated to be involved in the regulation of autophagy 31. Indeed, in MCF-7 cells, OHPg/PR-B cause a significant decrease in phosphorylated AKT cellular levels which is accompanied by a reduction in total AKT expression. These observations are in agreement with recent studies indicating that reactive oxygen species 41 or geldanamycin, an agent proposed for clinical testing in malignant melanoma, breast and prostate cancer 42,43, act by inducing AKT dephosphorylation and degradation. Specifically, degradation of active AKT *via* an ubiquitination-dependent pathway has been reported as a mechanism of regulation of AKT function in cell survival 41,43.

The importance of autophagy in regulating cell survival is only recently becoming realized. The link between autophagy and cancer appears to be complex and multifaceted although loss of autophagy is largely correlated with tumourigenesis 44,45 since several inducers of autophagy are tumour-suppressor genes 46. In this regard, PTEN has been widely recognized as a positive regulator of autophagy 47 through inhibition of AKT in glioma cells 48. Moreover, it has been reported an inverse relationship between AKT and UVRAG, a protein encoded by the ultraviolet irradiation resistance-associated gene, that represents a key factor in both autophagosome formation and maturation 49,50. Interestingly, we found that, in breast cancer cells, OHPg/PR-B/PTEN interplay causes a significant increase in UVRAG cellular protein levels which is because of the inhibition of AKT signalling. Indeed in the presence of a constitutive active AKT, OHPg treatment fails to up-regulate UVRAG expression. In this regard, a very recent article, suggested that the mechanism by which AKT inhibits UVRAG occurs at transcriptional level through binding to an unknown factor to form an inhibitory complex that binds at the UVRAG gene promoter region 31. Therefore, in our experimental model, AKT represents the molecular bridge linking the OHPg/PR-B up-regulation of PTEN with the appearance of the morpho-functional features of autophagy as monitored by flow cytometry analysis of acridine orange stained MCF-7 cells and TEM analysis evidencing the accumulation of autophagic vesicles, that was reversed by PR-B siRNA transfection.

**Figure 8:**
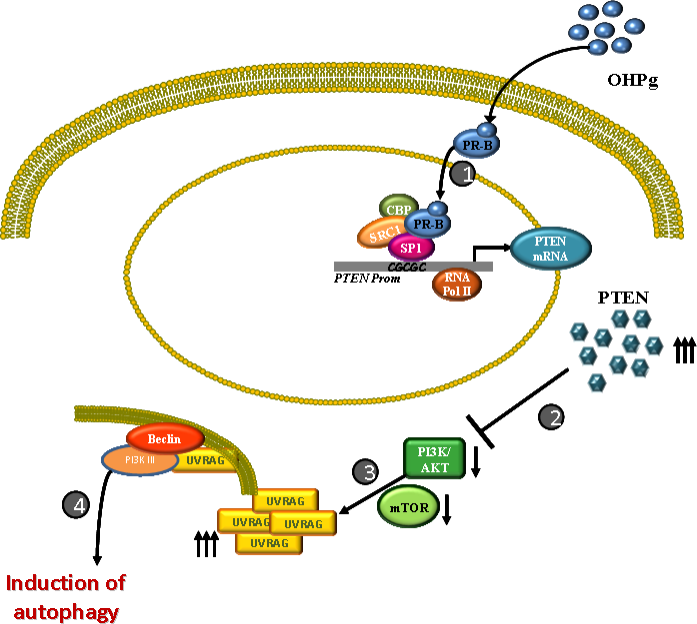
Proposed model for OHPg/PR-B/PTEN interplay in breast cancer cells. In the presence of OHPg, ligand-activated PR-B (1) binds to a specific SP1 motif within PTEN promoter gene triggering its activation. In turn, increased PTEN protein (2) inhibits AKT signalling thus allowing (3) UVRAG to be expressed contributing to the formation of the autophagosome vesicles.

On the basis of the results of this study, we propose a model for the inhibitory action of OHPg/PR-B/PTEN interplay on breast cancer cell survival and induction of the autophagy process. In this model, ligand-activated PR-B by inducing PTEN expression inhibits the AKT pathway which in turn lets UVRAG to be expressed thus allowing autophagosome formation (Fig. 8).

In conclusion, our results suggest that the pharmacological interference with OHPg/PR-B/PTEN transduction pathway deserves future studies. In fact, it could represent a novel useful therapeutic approach to be implemented in the conventional therapy of PR positive breast cancers, since it may sensitize cells to additional death inducing stimuli, potentiating autophagy response.

## Supporting Information

Figure S1 Western blot analysis of mTOR expression.

Figure S2 Western blot analysis of Beclin1 and PI3KIII expression.
