# Supplementary figures and images for: A novel functional interplay between Progesterone Receptor-B and PTEN, via AKT, modulates autophagy in breast cancer cells
Source: J Cell Mol Med. 2014 Sep 12;18(11):2252–65. doi: 10.1111/jcmm.12363 (PMC4224558; doi:10.1111/jcmm.12363)

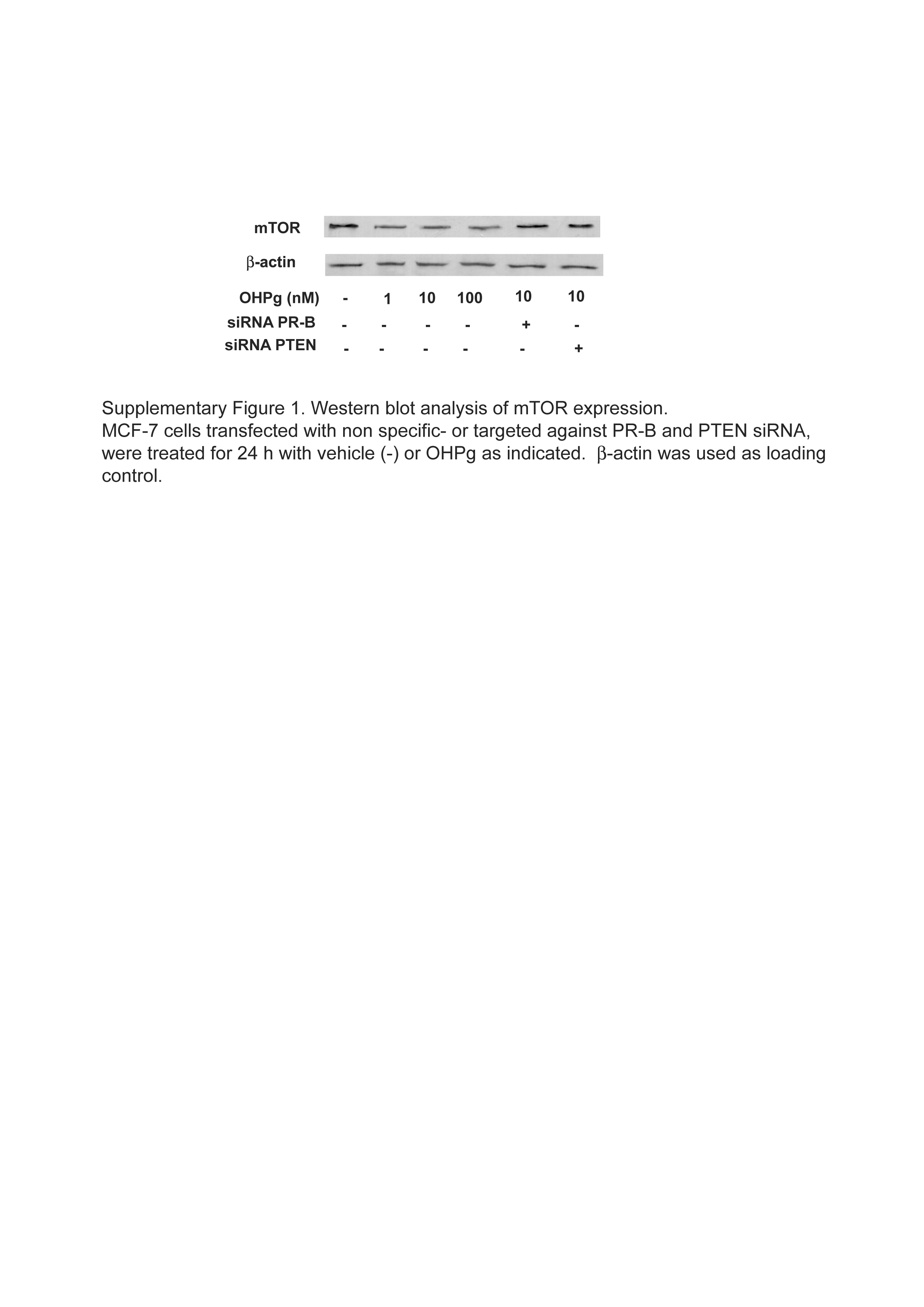

Supplement: Figure S1 — Western blot analysis of mTOR expression. [file jcmm0018-2252-sd1.tif]

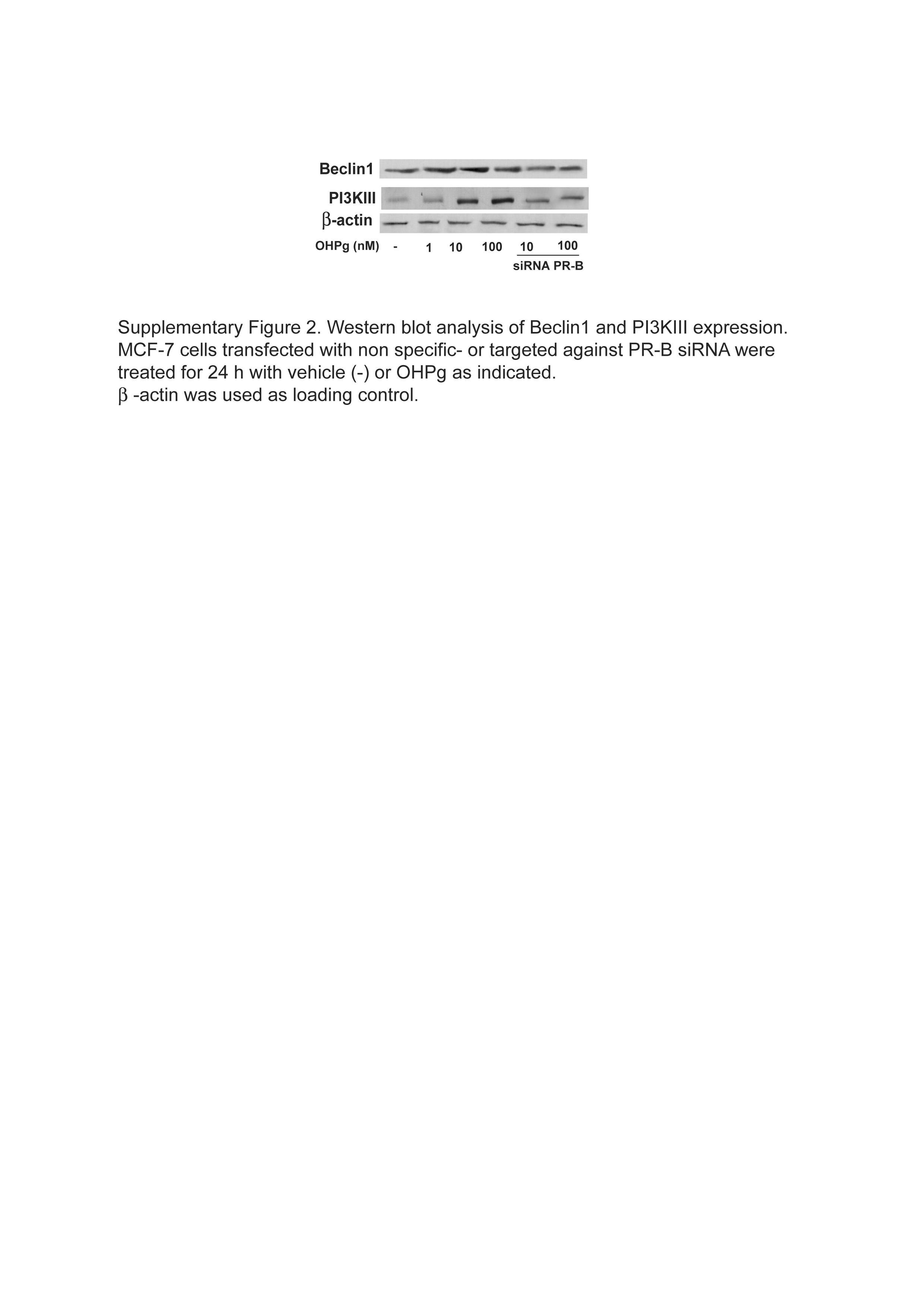

Supplement: Figure S2 — Western blot analysis of Beclin1 and PI3KIII expression. [file jcmm0018-2252-sd2.tif]
